# SenseBike: A New Low-Cost Mobile-Networked Sensor System for Cyclists to Monitor Air Quality and Automatically Measure Passing Distances in Urban Traffic

**DOI:** 10.3390/s25227099

**Published:** 2025-11-20

**Authors:** Andre Tenbeitel, Simone Arnold, Jens Rettkowski

**Affiliations:** Department of Electronics, University of Applied Sciences Dortmund, Sonnenstrasse 96, 44139 Dortmund, Germany; andre.tenbeitel@fh-dortmund.de (A.T.); jens.rettkowski@fh-dortmund.de (J.R.)

**Keywords:** environmental sensing, car distance detection, Particle Matter (PM) detection, wearables, open source

## Abstract

This study presents the development and validation of a low-cost, open-source sensor system for cyclists that automatically detects vehicle overtaking events while simultaneously monitoring air quality. The system integrates multiple ultrasonic sensors for autonomous overtaking detection and distance measurement with environmental sensors that record particulate matter, temperature, humidity, and GPS position. By combining these data streams, the system enables the analysis of correlations between traffic interactions and variations in particulate matter exposure under real-world cycling conditions. Test rides conducted in urban environments demonstrated that the system reliably identifies overtaking maneuvers and records corresponding environmental parameters. Elevated concentrations of particulate matter were observed during close vehicle passes and at traffic lights, highlighting moments of increased exposure to exhaust emissions. The automated detection mechanism eliminates the need for manual activation, ensuring complete and unbiased data collection. The modular design and energy-efficient operation of the system allow for flexible deployment in both mobile and stationary configurations. With its ability to objectively capture and relate safety and environmental data, the presented platform provides a foundation for large-scale field studies aimed at improving cyclist safety and understanding pollution exposure in urban traffic.

## 1. Introduction

In view of increasing urbanization and environmental awareness, cycling has become an essential component of sustainable urban mobility. With the growing number of cyclists in cities, issues related to safety and environmental exposure have gained increasing importance. Cyclists are frequently confronted with close overtaking maneuvers by motor vehicles and are simultaneously exposed to elevated levels of air pollution, particularly in mixed-traffic conditions [1,2,3,4,5]. These combined physical and environmental stressors pose significant risks to health and safety, yet they are often difficult to quantify objectively.

Existing research has addressed these issues from two separate perspectives. On the one hand, air quality can be monitored using mobile sensor networks and citizen-science approaches, providing valuable insights into particulate matter concentrations along urban routes [6,7]. On the other hand, studies on cyclist safety have investigated overtaking distances and driver behavior through manual or semi-automatic data collection methods [8,9]. Nevertheless, most systems remain either proprietary or require manual activation by the cyclist, which limits data completeness and reproducibility.

To bridge this gap, this study introduces a new low-cost and open-source sensor system that combines both aspects—environmental sensing and overtaking detection—within a single modular platform. The system is designed for autonomous operation, employing multiple ultrasonic sensors for automated vehicle detection and distance measurement while simultaneously recording environmental parameters such as particulate matter, temperature, and humidity. By integrating these datasets, the system enables a comprehensive assessment of how environmental and traffic conditions jointly affect cyclists in real-world urban environments.

The overarching objective of this research is not only to demonstrate the technical feasibility of such a system but also to explore its potential for identifying hazardous or highly polluted traffic situations. Ultimately, the resulting data may support urban planners and policymakers in developing evidence-based measures to improve cycling infrastructure and promote sustainable mobility.

The remainder of this paper is structured as follows: In the second section, related projects and existing technologies are listed to identify successful components of the sensor system. The third section presents the design and technical specifications of the system, focusing on its low cost and sustainability. In the fourth section, the implementation process and the software are discussed, followed by an analysis of initial results. The paper concludes with a summary of key findings and potential improvements.

### 1.1. Technology

The technological foundation of this study relies on two physical sensing principles: optical particle measurement for quantifying airborne particulate matter and ultrasonic time-of-flight measurement for determining distances to overtaking vehicles. Both techniques are suitable for compact and energy-efficient sensor integration in mobile environmental monitoring systems.

#### 1.1.1. Optical Particulate Matter Measurement

Particulate matter sensors typically operate based on the light-scattering principle. A laser or light-emitting diode illuminates an air sample within a defined chamber, and suspended particles scatter the incident light according to Mie theory [10]. The intensity of the scattered light $I_{s}$ depends on the particle diameter $d_{p}$, the refractive index *m*, and the scattering angle θ, and can be expressed as

(1) $$I_{s}\left(\theta \right)\propto I_{0}\;\frac{1}{r^{2}}\;{\left|S_{1}\left(\theta ,m,d_{p}\right)\right|}^{2}+{\left|S_{2}\left(\theta ,m,d_{p}\right)\right|}^{2},$$

where $I_{0}$ is the incident light intensity, *r* is the distance from the scattering volume, and $S_{1}$, $S_{2}$ are the Mie scattering amplitude functions. In practical sensor implementations, the scattered intensity is collected over a limited angular range determined by the photodetector geometry. By integrating the angular scattering signal, the particle number concentration *N* can be derived. The corresponding mass concentration $C_{PM}$ is then estimated using a calibration factor *k* as:

(2) $$C_{PM}=k\sum_{i}N_{i}\;\rho_{p}\;\frac{\pi d_{p,i}^{3}}{6},$$

where $\rho_{p}$ is the particle density and $d_{p,i}$ represents the effective particle diameter of class *i*. The mass concentration $C_{PM}$ can be determined for different particle size fractions, typically expressed as PM_1_, PM_2.5_, and PM_10_, corresponding to particles with aerodynamic diameters below 1 µm, 2.5 µm, and 10 µm, respectively. This optical method enables continuous, real-time measurement of these particulate matter concentrations in ambient air, making it well suited for mobile air-quality monitoring.

#### 1.1.2. Ultrasonic Distance Measurement

Ultrasonic distance measurement is based on the time-of-flight principle. An acoustic high-frequency pulse that cannot be heard by humans is emitted by a transducer and reflected by an object. The distance *d* between the sensor and the reflecting surface of the object is calculated from the travel time Δt between emission and echo detection as

(3) $$d=\frac{c\;\Delta t}{2},$$

where *c* is the speed of sound in air, which depends on the temperature *T* in °C according to

(4) $$c=331.3\frac{m}{s}\frac{1}{{}^{\circ}C}+0.606\frac{m}{s}\cdot T.$$

By using multiple ultrasonic transducers with different orientations, it is possible to distinguish between approaching and passing objects and to automatically detect overtaking events. This measurement principle provides a robust, low-cost, and contact-free method for determining vehicle proximity under varying environmental conditions.

### 1.2. Related Work

In this section, current research and developments in mobile air quality measurement and distance detection are discussed, providing an overview of relevant sensor technologies for these applications. Since there are multiple projects, only ones most relevant to this study are discussed in the following.

Section 1.2.1 describes systems available for measuring overtaking processes. Section 1.2.2 shows measurements of air quality. Section 1.2.3 compares these systems to the system presented in this paper.

#### 1.2.1. Systems for Measuring Overtaking Processes

The OpenBikeSensor measures the distance to overtaking vehicles and records these data along with GPS coordinates. As part of an open-source initiative, the system can be self-built, and users can contribute their data to a shared platform for analysis. The sensor consists of a handlebar-mounted module with a display and a button to manually trigger distance measurements during overtakes. However, the system requires user interaction to initiate the measurement process, which could distract cyclists [11].

The Space2Ride project, coordinated by TU Dresden, uses rear-facing bicycle lights equipped with dashcams to capture and analyze overtaking maneuvers. Its goal is to identify critical infrastructure that compromises cyclist safety. While the project aims to improve road safety through data analysis, little information is available about the hardware or system details [12].

The GARMIN varia system, which is a commercial solution, uses lidar and a camera to detect overtaking vehicles and alert the rider via a display. Although it provides warnings, it does not measure the distance between the cyclist and the vehicles. The system records video footage, but there is no integration for measuring or storing proximity data [13].

In [8], ultrasonic sensors were used to measure overtaking distance; however, the measurements were not triggered automatically.

In [9], an infrared laser was used for distance measurement; however, according to the definition applied in this work, this method cannot be considered low-cost.

#### 1.2.2. Air Quality Measurement Projects in Cycling

The Snuffelfiets project in the Netherlands uses SODAQ AIR (manufacturer: sodaq, Bussumerstraat 34, 1211 BL, Hilversum, The Netherlands) sensors to measure air quality along cycling routes. These sensors record particulate matter and transmit data using GSM. The initiative involves hundreds of cyclists, with data visualized on an online platform. However, the SODAQ AIR sensors are commercially available and not open-source, which limits flexibility for modification or anonymous use [14]. The ComPAIR project promotes citizen science for air quality monitoring, using the same SODAQ AIR sensors. It aims to involve citizens in data collection to influence environmental policies. This EU-funded initiative encourages public participation but relies on proprietary sensors [15].

In [6], the authors applied three low-cost sensors by using AlphaSense to measure particulate matter, NO_2_, and O_3_. The focus of their work was on evaluating the performance of these sensors and on using AI models to improve their calibration.

In [7], the authors used a smart citizen kit containing sensors for air quality and particulate matter measurements. Calibration was achieved by comparing the sensor data with official stationary monitoring stations, based on hourly integrated readings.

#### 1.2.3. Comparison of the Measurement Systems

A summary of the compared features is given in Table 1. While existing systems focus on either air quality monitoring or distance measurement, this project integrates both functionalities into a single, modular system. By combining overtaking vehicle detection with air quality monitoring, this expandable, low-cost solution offers a more comprehensive approach to improving cyclist safety and environmental awareness. The modular design also allows for future enhancements, making the system adaptable to evolving needs in urban cycling. Other important features include the fact that it is open-source, and the data is stored on a SIM card. This is important, as the data is easily accessible and data can be collected anonymously.

### 1.3. Research Questions

This study is guided by the following research questions:

1. How can multiple ultrasonic sensors be used to automatically detect overtaking events with sufficient accuracy and reliability under realistic cycling conditions?
2. Can measurements of various environmental parameters—such as particulate matter, temperature, and humidity—be utilized to identify traffic situations that are particularly stressful or harmful for cyclists?

By addressing these questions, this work seeks to enhance the understanding of cyclists’ exposure to both physical and environmental risks in urban traffic. The findings are expected to contribute to data-driven strategies for improving cycling safety and supporting sustainable urban mobility planning.

## 2. Materials and Methods

This section outlines the fundamental considerations in the development of the sensor system and its desired functions. Based on previous projects, successful components and technologies are analyzed to determine how they can be integrated into the sensor design. The focus is on creating a two-part sensor system, each with its own microcontroller, with both systems designed to operate independently.

**Figure 1:**
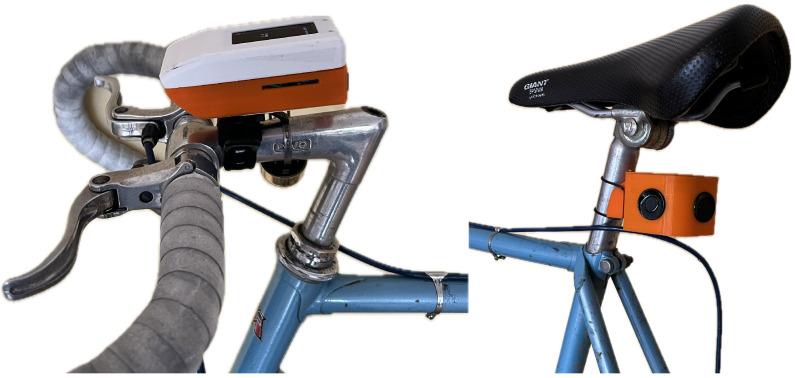
SenseFront and SenseBack Units mounted on a testbike.

The two sensor units are named SenseFront and SenseBack. SenseFront is the main sensor mounted on the handlebars, while SenseBack is the distance sensor mounted on the bike’s seat post (see Figure 1).

The SenseBack unit uses ultrasonic sensors based on the proven concept from the OpenBikeSensor project but with an improved vehicle detection. While OpenBikeSensor requires manual activation by the rider using a handlebar-mounted switch, the sensor presented in this paper is designed for automated activation. A second ultrasonic sensor detects approaching vehicles, which triggers the measurement process. This automation eliminates distractions for the rider and improves data accuracy and reliability. The data is transmitted wirelessly to SenseFront, further enhancing the system’s ease of use.

The SenseFront unit is designed to collect environmental data, such as temperature, humidity, and particulate matter, along with GPS coordinates. It features a display that shows the measured data in real-time, including warnings for approaching and overtaking vehicles detected by SenseBack. Additionally, the cyclists speed can be calculated based on GPS data. The environmental data is continuously synchronized with the GPS coordinates and stored on a microSD card, enabling a detailed spatial and temporal analysis of the environmental conditions along the cycling route.

### 2.1. Hardware

In the following, the hardware components used in the sensor system are described, focusing on their suitability for mobile use, energy consumption, size requirements, and resistance to external weather conditions.

- BOSCH BME680
  The BME680 is an integrated environmental sensor by Bosch Sensortec GmbH (Gerhard-Kindler-Strasse 9, Reutlingen, Germany) that measures temperature, humidity, air pressure, and volatile organic compounds (VOCs). It calculates an equivalent CO_2_ value (eCO_2_) based on VOC data through specialized algorithms, although it doesn’t directly measure CO_2_. The humidity can be measured but is not relevant for the planned analyses and therefore not discussed further.
- Sensirion SPS30
  The SPS30 is a compact, energy-efficient optical particulate matter (PM) sensor by Sensirion AG (Laubisruetisstrasse 50, Stäfa, Switzerland), capable of detecting particle sizes from 0.5 to 10 µm. It is well-suited for battery-powered mobile applications, making it ideal for this project. The sensor classifies particulate matter into PM1, PM2.5, PM4 and PM10 categories, which are transmitted via an I2C interface for further analysis.
  The influence of the gas flow rate has ben considered by the the contruction of the housing so the flow rate stays within the anticipated limits by the manufacturer (see Section 2.8).
- Ultrasonic Distance Measurement (JSN-SR04T)
  For detecting overtaking vehicles, the JSN-SR04T ultrasonic sensor (Elecrow, Nanchang Huafeng Industrial Park, CN Baoan District, Shenzhen, China) is employed due to its cost-effectiveness and accuracy in mobile environments. It is used in a dual-sensor configuration: one sensor measures the distance to overtaking vehicles, while the other detects approaching vehicles from behind.
- GPS Positioning (GROVE AIR530)
  The GPS sensor used is the GROVE AIR530 (Seeed Technology Co., Ltd. Tower B 1/F, Shanshui Building, Nanshan Yungu Innovation Industry Park, Liuxian Ave. No. 1183 CN 518055 Shenzhen, China), a compact and precise GNSS receiver capable of tracking multiple satellite systems. It is designed to perform well even in dense urban environments, and the data is transmitted via UART in NMEA 0183 format. Key data points include latitude, longitude, altitude, speed, and satellite quality metrics.
- Microcontroller ESP32
  Each sensor unit uses an ESP32 microcontrollerboard (espressif, Shangha, China), providing integrated Wi-Fi and Bluetooth capabilities. The microcontroller is versatile, supporting interfaces like SPI, I2C, and UART. Two different ESP32 models are used: the XIAO ESP32C3 for the SenseBack unit due to its compact size and integrated battery management, and the LILYGO T-Display-S3 for the SenseFront unit, which combines the microcontroller with an LCD for real-time data visualization.

### 2.2. Battery

Both sensor units, SenseFront and SenseBack, are powered by Li-Po (lithium-polymer) batteries with a nominal voltage of 3.7V. To align with the project’s low-cost and sustainable design goals, the batteries are sourced from disposable electronic cigarettes and reused in a second life approach. These electronic cigarettes typically contain small Li-Po batteries, which, despite being designed for single-use products, are capable of being repurposed for other applications. A study by the Bureau of Investigative Journalism [16] highlights the environmental impact of disposable electronic cigarettes, revealing that in the UK, approximately two vapes are discarded every second. This waste includes enough lithium annually to produce 1200 electric vehicle batteries. With only 50% properly recycled, lithium recovery remains a challenge, and improper disposal often causes battery fires in waste facilities, with 48% of UK waste fires linked to lithium batteries in disposable electronic cigarettes. In response to these challenges, the project adopts an environmentally conscious approach by reusing Li-Po batteries from electronic cigarettes. Batteries with capacities between 400 mAh and 700 mAh where used. A charging module was added to the battery to make the system rechargeable. Since the SenseFront and SenseBack units require only a single battery cell each, the complexity of battery management is minimized. There is no need for an external battery management system because the ESP32 microcontroller, used in both units, comes with integrated charging and battery management features, resulting in a simple design.

### 2.3. Housing

The housing is divided into two segments for optimal weight distribution, with components placed in both the upper and lower sections. The ESP32 and sensors are connected on a single circuit board to simplify assembly and create a more compact design. This configuration reduces the number of required pins on the ESP32 and allows for easier sensor interface management. The board is designed to facilitate easy replacement of the ESP32, enabling flexible communication options, such as LoRaWAN for long-distance wireless communication. The SenseBack unit does not require an additional circuit board, as its two identical sensors are directly connected to the microcontroller.

#### 2.3.1. SenseBack Design

**Figure 2:**
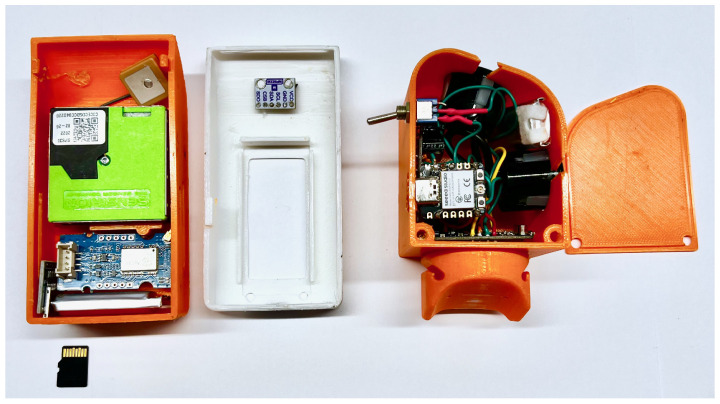
Components of SenseFront and SenseBack inside the device.

The SenseBack unit utilizes two JSN-SR04T ultrasonic sensors: one measures the distance to overtaking vehicles, while the second detects approaching vehicles to activate the main sensor only when needed, optimizing battery life. The sensors are positioned according to the manufacturer’s specifications, ensuring maximum detection range. The housing for SenseBack includes specific cutouts for the sensors and a modular mounting system that can accommodate varying seat post angles (see Figure 2). The unit is mounted using a rubber ring, which helps absorb vibrations and ensures easy detachment of the sensor while keeping the mount in place on the bicycle.

#### 2.3.2. SenseFront Design

The SenseFront housing is designed to meet several criteria, including ensuring proper airflow for particulate matter measurement and easy access to the USB-C charging port and MicroSD card without opening the case. The display is angled for easy readability, and heavy components are placed at the bottom to lower the center of gravity, increasing stability during vibrations caused by uneven surfaces. The housing features air inlets above the SPS30 sensor to avoid direct airflow, preventing inaccurate readings (see Figure 2). The SenseFront unit is attached to the handlebars using a front light mount, with an adapter providing a stable connection between the sensor and the handlebar mount.

### 2.4. Software

The SenseFront and SenseBack units follow structured workflows to perform their functions. The SenseFront unit initializes sensors, sets up ESPNOW communication, and handles continuous data recording. If any sensor fails to initialize, an error message is displayed. The SenseBack unit handles vehicle detection and distance measurement, transmitting the data back to SenseFront via ESPNOW. The vehicle detection process on SenseBack operates in three states:

- IDLE :
  No vehicle is detected, and the distance sensor is inactive.
- APPROACHING:
  A vehicle is detected within 250 cm, activating the distance sensor.
- PASSING:
  The distance to overtaking vehicles is recorded until no more vehicles are detected, switching back to IDLE.

**Figure 3:**
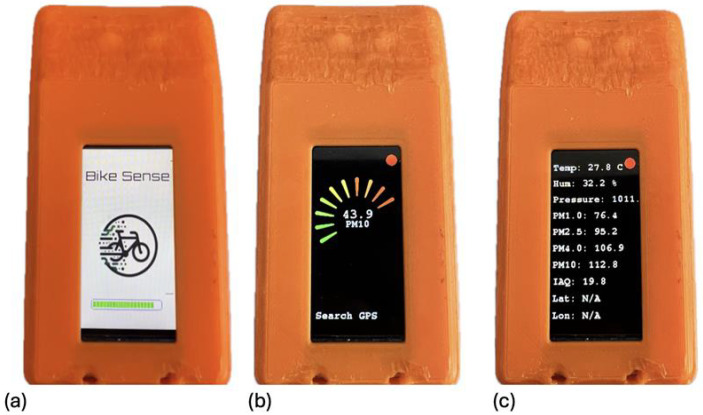
(**a**–**c**) Display screens to visualize the data and the systems status.

Both units can enter a Deep Sleep mode to conserve energy e.g., during cycling breaks, activated by holding both buttons for two seconds, allowing sensors to remain calibrated and enabling faster GPS signal acquisition upon wake-up reactivation is achieved similarly by pressing a button for two seconds. The SenseFront unit features a user-friendly display system that provides real-time information to the cyclist without causing distractions. After initialization, the display shows a main dashboard with critical data such as particulate matter levels and the current speed, measured by the GPS sensor. The air quality data is visualized with a circular indicator that changes color from green to red, depending on the detected levels of particulate matter, signaling increased pollution. A warning of approaching vehicles is given automatically. A secondary screen, accessible through the device’s two-button interface, shows detailed sensor data including temperature, humidity, pressure, and other measurements, allowing users to get a comprehensive overview without overloading the main display. This layered approach to displaying data ensures that essential information is easily accessible while minimizing distractions during cycling. Examples for display screens are shown in Figure 3.

Due to the large amount of sensor data, continuously displaying all measurements is not practical, as it would result in an overloaded interface. To address this, a display menu is implemented, controlled via the two buttons on the ESP32. Upon startup, all sensors are initialized, and this process is visualized on the display with a loading bar as shown in Figure 3a. If any sensors fail to initialize, an error message is displayed. The main screen focuses on displaying particulate matter levels as both numerical values and graphical indicators (see Figure 3b,c). A circular indicator shows the particulate matter levels with color-coded bars, ranging from green to red, with red indicating elevated pollution levels. The current speed, calculated via the GPS module, is also shown. Additionally, a 1 Hz blinking banner alerts the cyclist when a vehicle is approaching from behind. A second screen can be accessed to view additional sensor data, such as temperature, humidity, and pressure, ensuring a complete overview without cluttering the main display. This design prioritizes essential information during the ride, allowing for safer and more effective monitoring.

### 2.5. Calibration

To ensure accurate and reliable measurements, the calibration and functionality of the sensors are verified under controlled conditions.

### 2.6. Ultrasound Distance Calibration

**Figure 4:**
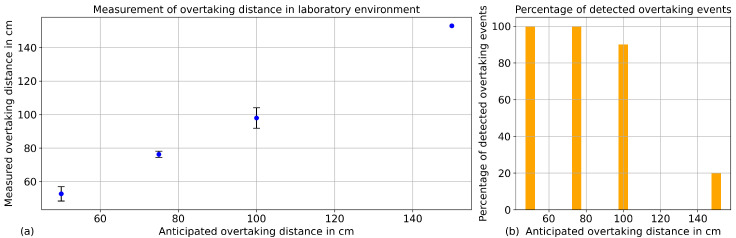
Results of laboratory tests of overtaking events. (**a**) measured overtaking distances. (**b**) percentage of detected overtaking events at different distances.

To ensure accurate distance measurements, the ultrasonic sensor was calibrated prior to data collection. A controlled test setup was used to evaluate the sensor’s performance at low velocities, simulating realistic overtaking scenarios. These tests included both a moving vehicle passing a stationary bicycle and a bicycle passing a stationary vehicle. In addition to the laboratory tests, a field test was conducted under real-world conditions to validate the sensor system in dynamic traffic environments. The laboratory tests demonstrated that the angle between the two sensors—one responsible for detecting the overtaking event and the other for measuring the distance—is critical for achieving reliable measurements. In the laboratory experiments, almost all overtaking events at distances of 50 cm, 75 cm, and 100 cm were successfully detected with good accuracy (see Figure 4). These distances are particularly relevant, as close overtaking maneuvers pose the greatest safety risk. Detecting vehicles at larger distances would require an adjustment of the sensor’s viewing angle. Similarly, in the field tests, the initiation of overtaking events was detected with high reliability. Only in situations where multiple vehicles were driving very closely behind one another, or diagonally offset across multiple lanes, could the system not distinguish them as individual vehicles; instead, these cases were interpreted as a single overtaking event.

### 2.7. Temperature Calibration

**Figure 5:**
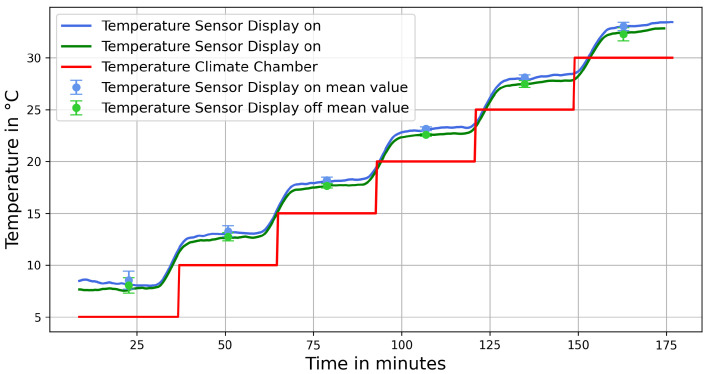
Temperature calibration in Climate chamber. The temperature measurement must be corrected by an offset because the electronics generates additional heat.

The temperature sensor is subjected to a temperature cycle in the climate chamber with constant humidity. The temperature is varied in steps from 5 °C to 30 °C, held at each level for 30 min to allow the sensor to acclimate (see Figure 5). This cycle is repeated twice, once with the display turned off and once with the display set to 100% brightness using pulse-width modulation. The comparison of the two temperature curves shows that the display affects the sensor’s temperature readings and that the electronics themselves generate additional heat.

To compensate for this heat effect and the temperature sensor’s offset, a software correction was implemented that applies a negative temperature offset of 3 °C when the display is on, and a slightly smaller offset when the display is off. After applying this correction, the deviation from the set temperature is reduced to less than 1 °C, which is considered sufficiently accurate.

### 2.8. Particulate Matter Measurement

The particulate matter sensor used in this study is factory-calibrated, eliminating the need for additional calibration. A detailed study of the performance and particle size-selectivity can be found in [17] and is therefore not studied in this paper.

The housing of the SenseFront module was specifically designed to reduce external airflow and ensure that the sensor operates within the manufacturer’s specified limits for air velocity and flow conditions. To verify the sensor’s stability, measurements were performed under constant temperature and humidity inside a climate chamber.

**Figure 6:**
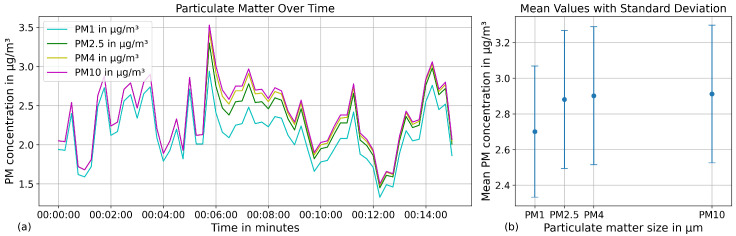
(**a**) Temporal fluctuations in particulate matter concentrations during a 15 min measurement period. (**b**): Measurement deviations of particulate matter concentration observed over a one-minute interval.

A measurement conducted in a climate chamber at constant particulate matter (PM) concentration showed that the readings exhibited minor fluctuations but remained stable over a longer period (see Figure 6).

**Figure 7:**
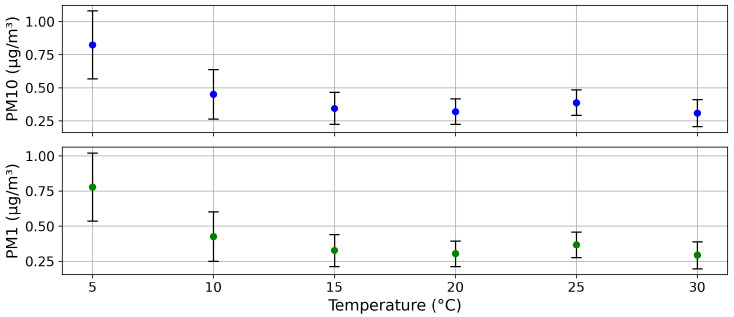
Temperature dependency of particulate matter measurement over a temperature range of 5 to 30 °C.

Furthermore, temperature variations within a range of 5 °C to 30 °C had negledgeable influence on the particulate matter measurements (see Figure 7).

These results confirm the sensor’s stability under controlled environmental conditions and provide a reliable baseline for subsequent experiments investigating the effects of varying humidity on particulate matter readings.

Accurate airflow management is essential for maintaining the measurement precision of optical particulate matter sensors such as the Sensirion SPS30. The sensor incorporates an internal fan that generates a defined and steady volumetric airflow through the measurement chamber. This internal flow is part of the factory calibration and ensures consistent particle sampling and optical scattering conditions. External air movements—such as wind or pressure fluctuations caused by vehicle motion—can interfere with this internal flow and lead to biased or unstable readings.

To prevent excessive airflow and crosscurrents, the SenseFront housing was engineered to decelerate and diffuse the incoming air stream. The inlet geometry minimizes direct wind exposure, allowing the sensor to operate within the manufacturer’s recommended airflow parameters (typically below 1 m/s). This design ensures that the internal fan remains the dominant driver of the sampling airflow, thereby preserving calibration integrity and ensuring accurate particulate matter measurements even under dynamic outdoor conditions.

### 2.9. GPS Accuracy and Positioning

The accuracy of the GPS module was evaluated by comparing the recorded positions with known reference coordinates. The measurements showed an average positional deviation of 6.93 m, which is well within the acceptable range for mobile environmental sensing applications. The cold start time of the GPS—defined as the duration from power-on to the first successful satellite fix—averaged 46 s across four independent trials.

### 2.10. Energy Consumption

Energy consumption was evaluated for each component of the sensor system. The ESP32 microcontroller exhibited a current draw between 60 and 170 mA, depending on whether the Wi-Fi module and display were active. When all sensors were operating and data were transmitted via ESP-NOW during mobile measurements, the average current consumption was approximately 301.18 mA.

For stationary operation, with the display and GPS module disabled, the average current consumption decreased to 149.55 mA. Between measurement cycles, the system automatically entered a deep-sleep mode, reducing the current draw to as low as 2.11 mA. This power management strategy significantly improves the system’s energy efficiency and extends the operational lifetime, particularly when powered by second-life batteries.

## 3. Results and Discussion

This section presents the results obtained from the mobile sensor system during several test rides in urban traffic. The analysis focuses on two main aspects: (i) the automated detection and evaluation of vehicle overtaking maneuvers, and (ii) the assessment of particulate matter concentrations to characterize cyclists’ exposure to air pollution under real-world conditions. Together, these datasets provide valuable insights into the complex interaction between traffic dynamics and air quality in urban cycling environments.

### 3.1. Overtaking Distance Analysis

**Figure 8:**
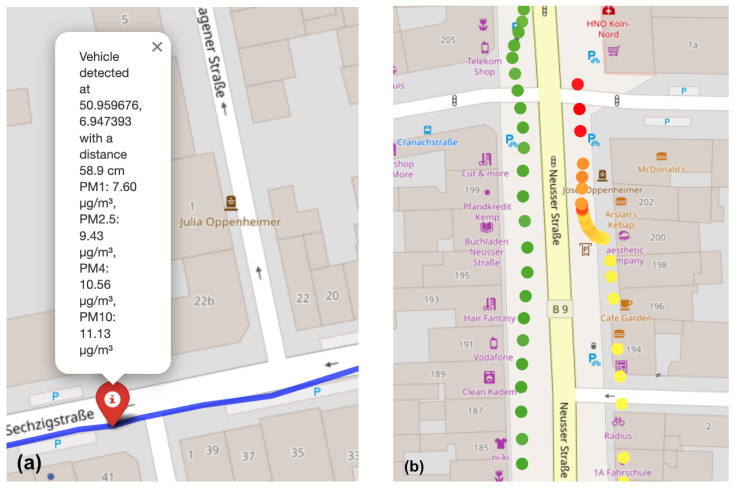
(**a**) Automatically initialized overtaking distance measurement event. (**b**) PM10 data collected during a test ride in the Cologne area, showing high particulate matter exposure at a traffic light, red indicating distances below 75 cm. yellow indicating distances between 75–150 cm and green indicating distances above 150 cm.

The data collected during the test rides (see an example in Figure 8) provided detailed information about vehicle overtaking distances in different traffic situations.

The analysis revealed that 66.9% of the vehicles did not maintain the legally required minimum distance when overtaking cyclists, with 36.7% of all recorded maneuvers occurring at dangerously close distances below 75 cm. The automatic vehicle detection and distance measurement system functioned reliably overall, accurately identifying most overtaking events. Inaccuracies occurred in specific cases involving vehicles moving back and forth, or when multiple vehicles overtook very close to each other, causing them to be counted as a single overtaking event. These deviations suggest that further calibration or the integration of additional ultrasonic sensors could enhance the robustness of the detection system. Alternatively, improved data processing algorithms may further increase classification accuracy.

A notable observation from the combined dataset is the relationship between vehicle proximity and local air quality degradation which was best visible at traffic light stops (see Section 3.2).

### 3.2. Particulate Matter Analysis

The particulate matter data collected during the test rides provided additional insights into the air quality conditions encountered by cyclists in various urban scenarios. The highest concentrations of particulate matter were observed in areas with dense traffic, particularly near intersections and traffic lights where cyclists were forced to stop close to idling vehicles. In these situations, short-term peaks in particulate matter were recorded, indicating acute exposure to exhaust-related fine particles. Peaks in PM_10_ concentrations were also detected, likely caused by coarse particles resuspended by vehicle motion.

Despite these localized peaks, the average particulate matter concentrations across the entire route remained below the legally mandated limits. Nevertheless, the transient spikes observed during traffic stops are concerning, especially since cyclists’ elevated breathing rates during physical activity can increase pollutant intake and associated health risks.

**Figure 9:**
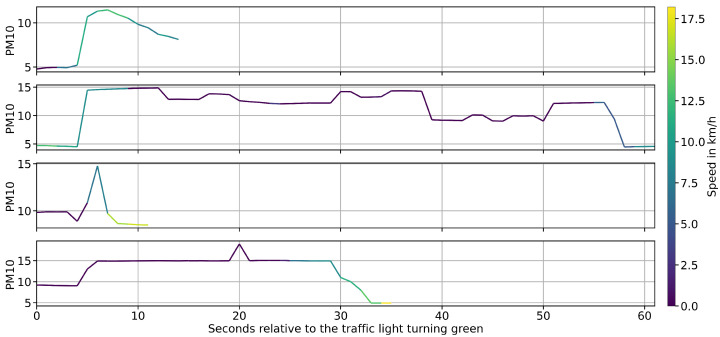
Comparison of PM_10_ concentration profiles at several traffic light stops. Peak concentrations are observed at the onset of the green phase, when vehicles accelerate and pass the cyclist.

Figure 9 illustrates the PM_10_ concentration profiles recorded at exemplary traffic light stops. The highest particulate matter levels occurred at the start of the green phase, when vehicles accelerated past the cyclist. These findings underscore the direct link between traffic behavior and short-term air pollution exposure. In combination with the overtaking data, the results demonstrate how the developed system enables a comprehensive assessment of both traffic safety and environmental stressors in urban cycling environments.

## 4. Conclusions

This study successfully achieved its primary objectives: the development of a low-cost, mobile, and modular sensor system capable of simultaneously monitoring air quality and traffic safety in urban cycling environments. The system combines automated vehicle detection and overtaking distance measurement with environmental sensing, offering a comprehensive tool for assessing cyclists’ exposure to both physical and environmental risks. Core design goals—including the use of second-life batteries, an open-source architecture, and a modular hardware structure—were fully realized, ensuring sustainability, accessibility, and adaptability for future research needs.

The prototype demonstrated reliable performance in real-world conditions, continuously recording particulate matter, temperature, humidity, and GPS data. These measurements were visualized on a map, allowing for the identification of specific route segments where overtaking events occurred and their correlation with local air quality levels. This capability provides a holistic understanding of the urban cycling environment and highlights areas where cyclists experience increased safety risks or higher pollution exposure.

When several vehicles were driving very close together or diagonally across multiple lanes they could not be resolved as individual vehicles, resulting in a single detected overtaking event. To address this limitation, combining the system with an additional sensor or employing a multi-sensor fusion approach should be considered.

The modular system design enables straightforward integration of additional sensing units and communication interfaces. Future extensions may include advanced wireless communication technologies, such as LoRaWAN, to facilitate data transmission over long distances, as well as supplementary sensors for measuring gaseous pollutants like NO_x_ and O_3_. These enhancements would broaden the system’s analytical scope and improve its suitability for large-scale environmental and traffic studies.

Because each data point is georeferenced, it is also possible to distinguish between sections with and without dedicated cycling infrastructure by aligning the measurements with mapping resources such as OpenStreetMap. This feature enhances the value of the collected data for evidence-based urban planning and policy development.

In summary, the developed system demonstrates the feasibility of combining automated overtaking detection with environmental monitoring in a single, scalable platform. Its modular architecture and robust data acquisition capabilities make it well suited for future field studies aimed at understanding cyclists’ exposure under diverse traffic and environmental conditions. The results of such deployments could support data-driven strategies for improving cycling safety, optimizing urban air quality, and advancing sustainable mobility planning.

## Figures and Tables

**Table 1:** Comparison of Related Projects.

	This Project	OpenBike Sensor [11]	Space2- Ride [12]	GARMIN VARIA [13]	Snuffelfiets [14]	ComPAIR [15]
VDD ^1^	X	X	X			
VD ^2^	X		X	X		
OS ^3^	X	X				X
EDC ^4^	X				X	X
GPS ^5^	X		X		X	X
ADC ^6^	X	X				

^1^ vehicle distance detection; ^2^ vehicle detection; ^3^ open-source; ^4^ environmental data collection; ^5^ GPS positioning; ^6^ anonymous data collection.

## Data Availability

The original contributions presented in this study are included in the article. Further inquiries can be directed to the corresponding author(s).
